# Metabolic engineering of narrow‐leafed lupin for the production of enantiomerically pure (−)‐sparteine

**DOI:** 10.1111/pbi.14509

**Published:** 2024-11-22

**Authors:** Davide Mancinotti, Ting Yang, Fernando Geu‐Flores

**Affiliations:** ^1^ Section for Plant Biochemistry and Copenhagen Plant Science Centre, Department of Plant and Environmental Sciences University of Copenhagen Frederiksberg Denmark

**Keywords:** Biosynthesis, FIND-IT, knockout, lupin alkaloids, metabolic engineering, sparteine

## Abstract

The protein crops known as lupins have been bred to accumulate low levels of antinutritional alkaloids, neglecting their potential as sources of valuable metabolites. Here, we engineered narrow‐leafed lupin (NLL) to accumulate large amounts of a single alkaloid of industrial interest called (−)‐sparteine. While (−)‐sparteine is recognized as a key auxiliary molecule in chiral synthesis, its variable price and limited availability have prevented its large‐scale use. We identified two enzymes that initiate the conversion of (−)‐sparteine to a variety of alkaloids accumulating in NLL. The first one is a cytochrome P450 monooxygenase belonging to family 71 (CYP71D189), and the second one is a short‐chain dehydrogenase/reductase (SDR1). We screened a non‐GMO NLL mutant library and isolated a knockout in CYP71D189. The knockout displayed an altered metabolic profile where (−)‐sparteine accounted for 96% of the alkaloid content in the seeds (GC–MS basis). The (−)‐sparteine isolated from the mutant seeds was enantiomerically pure (99% enantiomeric excess). Apart from the altered alkaloid profile, the mutant did not have any noticeable phenotype. Our work demonstrates that (−)‐sparteine is the precursor of most QAs in NLL and expands the current uses of NLL as a crop.

## Introduction

Lupins (*Lupinus* spp.) are promising protein crops that accumulate up to 40–50% protein in the seeds. Wild lupins accumulate large amounts of quinolizidine alkaloids (QAs), which can cause anticholinergic poisoning, including blurry vision, tachycardia, and sometimes death (Rietjens and Eisenbrand, 2023). The modern domestication of lupins started only a century ago with the development of “sweet” lines with low QA content. The addition of key agronomic traits enabled the creation of the sweet varieties commonly available today, with three species predominating: narrow leafed lupin (*L. angustifolius*, NLL), white lupin (*L. albus*), and yellow lupin (*L. luteus*) (Cowling *et al*., 1998). The popularization of sweet lupins and the focus on maintaining low alkaloid levels (Frick *et al*., 2017) have directed attention away from the potential use of lupins as sources of individual QAs with interesting pharmaceutical and chemical properties. In this work, we used state‐of‐the‐art plant breeding technology to engineer the native biosynthetic pathway of QAs in NLL for producing commercially valuable (−)‐sparteine, a key molecule in the field of asymmetric chemical synthesis.

Asymmetric synthesis is the synthesis of chiral molecules using methods that favour the formation of a specific enantiomer/diastereomer. This is particularly relevant for the pharmaceutical industry, as different enantiomers/diastereomers typically have different biological activities. Sparteine is one of the most versatile auxiliary molecules used in asymmetric synthesis. Specifically, complexes between sparteine and lithium have proven unrivalled for common asymmetric procedures such as deprotonations, substitutions, carbometalations, and *orto‐*metalations (Hoppe and Hense, 1997; Chuzel and Riant, 2005; Kizirian, 2010). Originally, only (−)‐sparteine was commercially available, enabling the asymmetric synthesis of just one of two possible products in each case. This led to the development of a synthetically accessible molecule with the expected functionality of (+)‐sparteine, which became known as the (+)‐sparteine surrogate (Dearden *et al*., 2002). Commercial (−)‐sparteine remained cheap throughout the 2000s (Jones, 2008); however, for reasons that remain unclear, it later became expensive and, at times, fully unavailable (Lowe, 2010; Ritter, 2017; Lowe, 2022). The fluctuations in price and availability motivated the development of a (−)‐sparteine surrogate (Firth *et al*., 2014) as well as an improved synthetic route for (−)‐sparteine itself (Firth *et al*., 2018). However, the multistep nature of these protocols (8 and 10 steps, respectively) (Firth *et al*., 2018) has prevented their adoption by the wider community.

**Figure 1:**
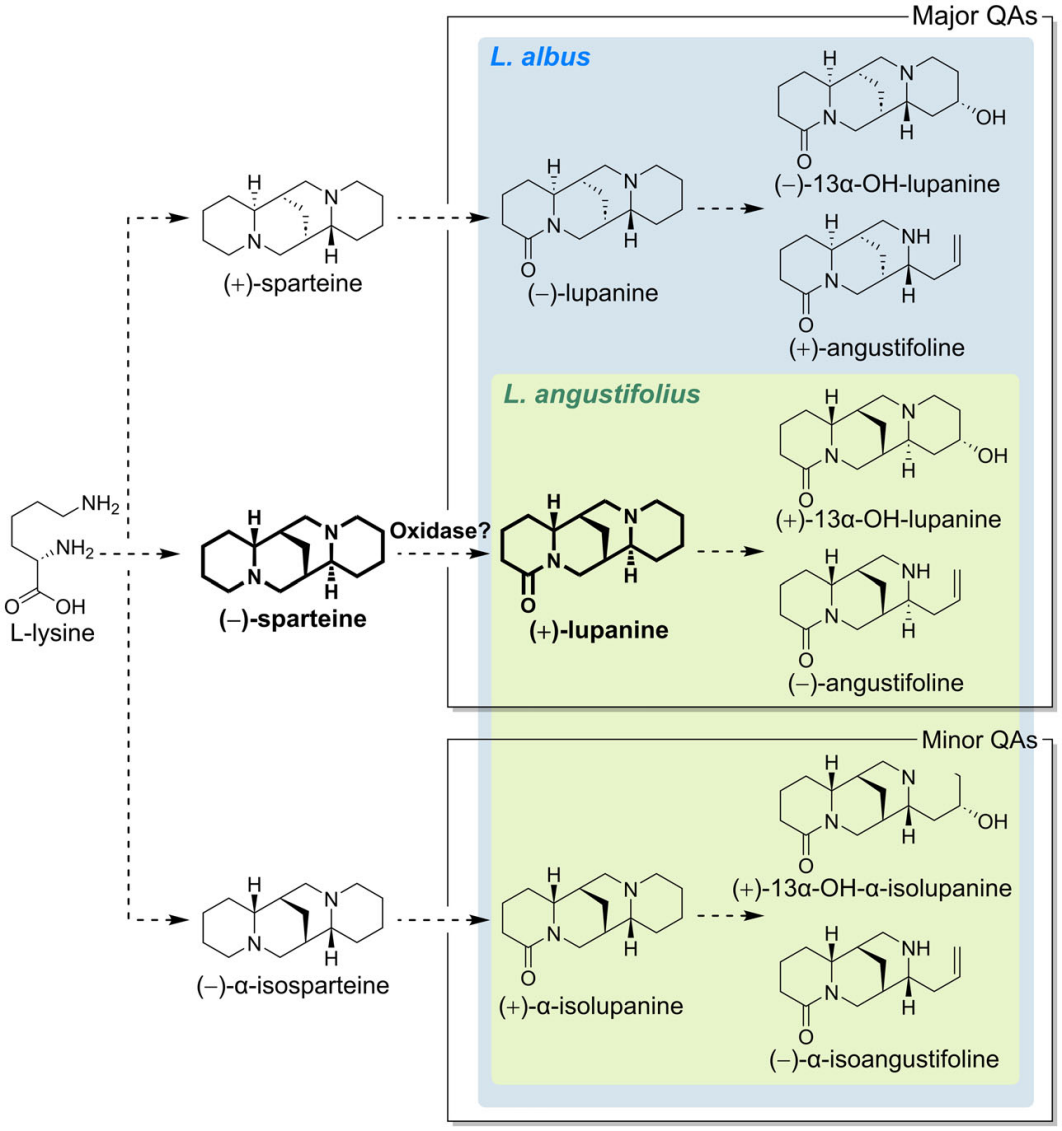
QA occurrence and biosynthesis in two common lupin crops, white lupin (*Lupinus albus*) and narrow‐leafed lupin (NLL, *Lupinus angustifolius*). Overall biosynthetic proposal based on the backbone structure of the QAs found in each species. The proposed two‐step enzymatic oxidation of (−)‐sparteine to (+)‐lupanine (in bold) is the subject of this study. In addition to the QAs shown above, white lupin also accumulates multiflorine and derivatives (Kroc *et al*., 2017), and both species accumulate esterified derivatives of 13α‐OH‐lupanine (Lee *et al*., 2006; Wink *et al*., 1995).

Lupins typically accumulate complex mixtures of QAs (Wink *et al*., 1995), including bicyclic, tricyclic, and tetracyclic ones. Structurally, sparteine is the simplest tetracyclic QA (see structure in Figure 1). The enantiomeric purity of lupin‐derived QAs is species‐dependent. For example, the tetracyclic QA lupanine occurs in white lupin (*L. albus*) as a near‐racemic mixture (Davis, 1897), while the same QA in narrow‐leafed lupin (NLL, *L. angustifolius*) seems to occur exclusively as the dextrorotatory form (Davis, 1897) (Figure 1). Since the seeds of “bitter” (high‐QA) white lupin are consumed as snacks in Southern Europe upon extensive alkaloid removal, chemists have developed methods for the chiral resolution of lupanine and its subsequent reduction to sparteine (Clemo *et al*., 1931; Maulide *et al*., 2017; Przybył and Kubicki, 2011). Interestingly, several wild North American lupins predominantly accumulate (−)‐sparteine, including *L. babiger* (Couch, 1932) and *L. montanus* (Regla *et al*., 2019). Outside the *Lupinus* genus, few species are known to display similar characteristics, in particular, scotch broom (*Cytisus scoparius*, also known as *Sarothamnus scoparius*) (Cionga *et al*., 1957; Gustowski and Kroszczynski, 1960), which appears to have been the source of commercial (−)‐sparteine throughout the 1990s and 2000s (Ritter, 2017). The extent to which any of these (−)‐sparteine‐accumulating plants can become a reliable commercial source remains unclear, as these are all wild species not optimized for production and likely prone to large seasonal variation.

Our group is currently studying the biosynthesis and transport of QAs in NLL, which is the most commonly cultivated lupin species. NLL does not normally accumulate (−)‐sparteine but accumulates at least eight related QAs, three of which are efficiently transported to the seeds (Frick *et al*., 2017; Otterbach *et al*., 2019) (up to ~3% by weight in bitter lines (Kamel *et al*., 2016)). By comparing the backbone structures of the major QAs found in NLL (Otterbach *et al*., 2019; Wink *et al*., 1995), we hypothesized that (−)‐sparteine may be a common biosynthetic intermediate and that a 2‐step enzymatic oxidation would transform it into the abundant (+)‐lupanine (Figure 1). Here, we report the discovery of two different enzymes sequentially catalysing this oxidation as well as the generation of an engineered NLL line that can be used for the industrial production of (−)‐sparteine.

## Results and discussion

To verify the previous reports that (+)‐lupanine from NLL was enantiomerically pure (Davis, 1897), we subjected plant extracts to analysis by liquid chromatography coupled to mass spectrometry (LC–MS) using a chiral column. Comparison to authentic standards confirmed that both leaves and seeds accumulated (+)‐lupanine exclusively (Figure S1). This suggests that most of the metabolic flux towards QAs in NLL goes through (−)‐sparteine.

**Figure 2:**
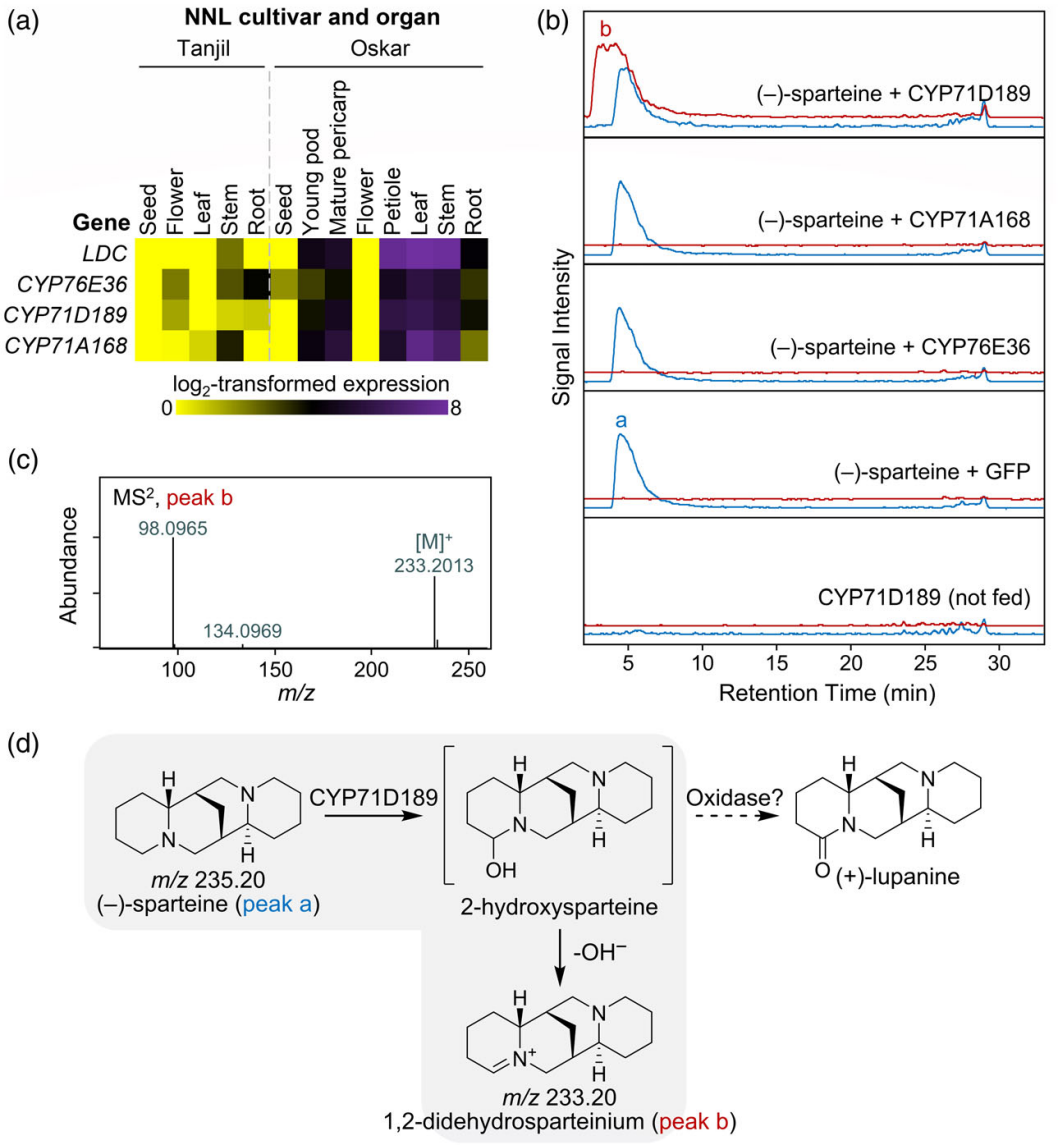
CYP71D189 is a putative sparteine 2‐hydroxylase. (a) Gene expression heatmap of the three candidate oxidases CYP76E36 (LOC109338642), CYP71D189 (LOC109360201), and CYP71A168 (LOC109357725) in relation to the known QA pathway enzyme LDC (LOC109327937). Colours represent expression values in transcript per million (TPM). (b) LC–MS analysis of extracts of *N. benthamiana* leaves expressing CYP71D189 or two other oxidase candidates at 9 days post infiltration (dpi) following feeding with (−)‐sparteine at 4 dpi. Peak a corresponds to (−)‐sparteine, and peak b corresponds to a didehydrosparteinium ion. Unfed leaves expressing CYP71D189 and fed leaves expressing GFP were included as controls. The traces are representative extracted ion chromatograms corresponding to sparteine ([M + H]^+^, *m/z* 235.22 ± 0.01, blue trace) and 1,2‐didehydrosparteinium (M^+^, *m/z* 233.20 ± 0.01, red trace). The traces are slightly offset to aid visualization of the otherwise overlapping peaks. (c) ESI+ CID MS^2^ mass spectrum (24.0 eV) of putative 1,2‐didehydrosparteinium from CYP71D189‐expressing *N. benthamiana* leaves fed with (−)‐sparteine. (d) Proposal for the CYP71D189‐catalysed hydroxylation in the absence of a subsequent enzyme‐catalysed dehydrogenation (grey background).

As genes involved in a given specialized metabolite pathway tend to be co‐expressed (Delli‐Ponti *et al*., 2021), we used available transcriptomics datasets (Kamphuis *et al*., 2015; Yang *et al*., 2017) to select three oxidase candidates whose expression patterns resembled those of the known QA pathway enzyme lysine decarboxylase (LDC) (Figure 2a, Table S1). We named the candidates CYP76E36 (LOC109338642), CYP71A168 (LOC109357725), and CYP71D189 (LOC109360201) based on their inclusion in the repository of cytochromes P450 maintained by Dr. David R. Nelson (University of Tennessee, Memphis, TN, USA) (Nelson, 2018). We cloned the coding sequences from cDNA and expressed them individually in *Nicotiana benthamiana* via agroinfiltration. Similar to the negative control (GFP), leaves expressing CYP76E36 and CYP71A168 seemed not to be able to metabolize the separately infiltrated (−)‐sparteine. By contrast, leaves expressing CYP71D189 produced a putative didehydrosparteinium ion upon infiltration with (−)‐sparteine (Figure 2b–d). We speculated that CYP71D189 was able to hydroxylate (−)‐sparteine at position 2, and that, in the absence of a second, dedicated oxidase, the adjacent nitrogen eliminated the hydroxyl group spontaneously (either *in planta* or in the acidic extract (Ebner *et al*., 1991)) (Figure 2d).

**Figure 3:**
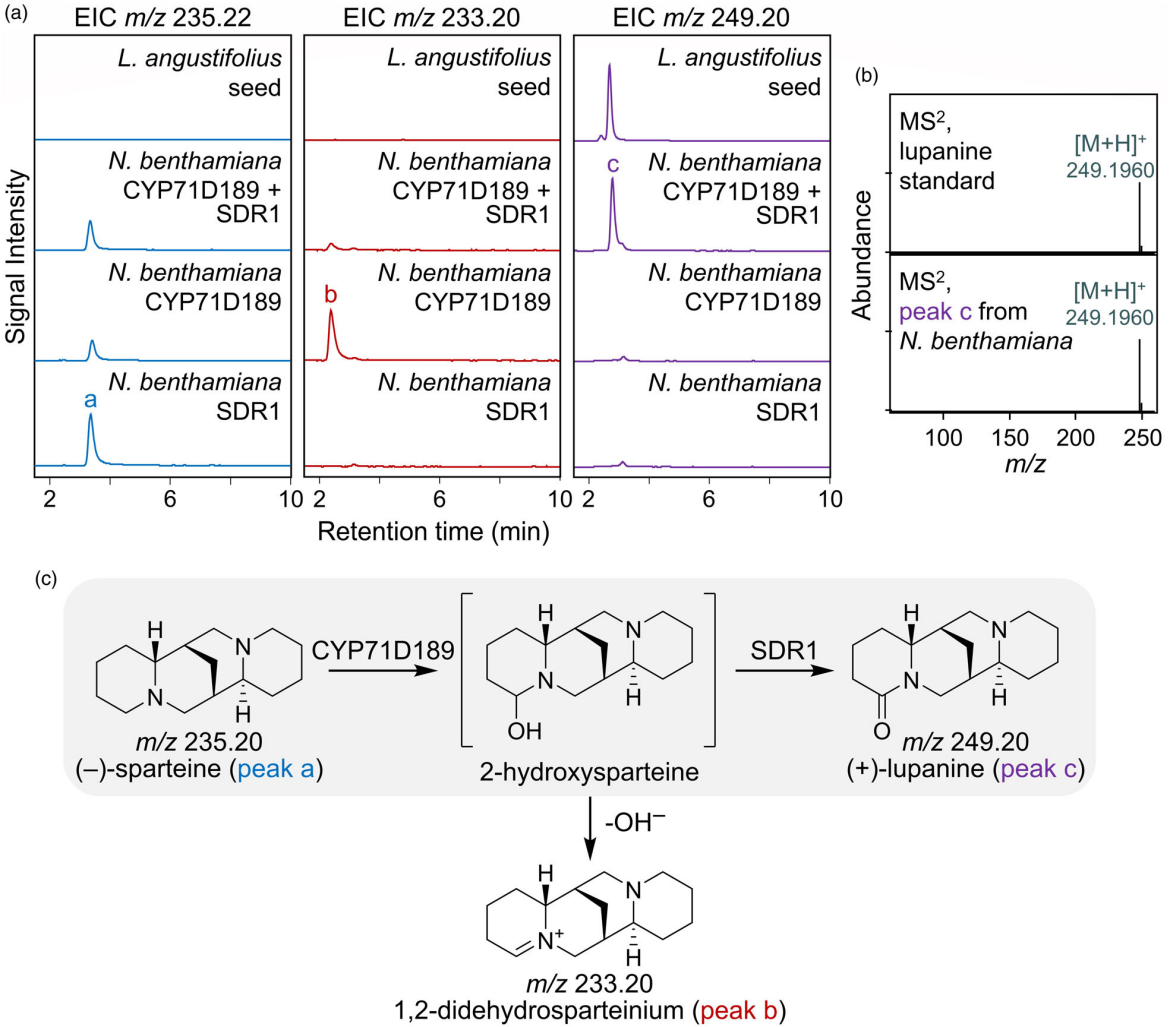
Co‐expression of CYP71D189 and SDR1 enables production of (+)‐lupanine from (−)‐sparteine in *Nicotiana benthamiana*. (a) LC–MS analysis of extracts of *N. benthamiana* leaves expressing CYP71D189 and SDR1 at 8 days post infiltration (dpi) following feeding with (−)‐sparteine at 4 dpi. The traces are representative extracted ion chromatograms corresponding to sparteine ([M + H]^+^, *m/z* 235.22 ± 0.01, left column), 1,2‐didehydrosparteinium (M^+^, *m/z* 233.20 ± 0.01, middle column), and lupanine ([M + H]^+^, *m/z* 249.20 ± 0.01, right column). Respective traces from the analysis of NLL seed extracts are also shown for comparison. Peak labels correspond to the compounds shown in panel c. (b) ESI+ CID MS^2^ (24.7 eV) mass spectrum of lupanine produced in *N. benthamiana* leaves in comparison to a commercial standard. (c) Proposed pathway for the stepwise oxidation of (−)‐sparteine, first to 2‐hydroxysparteine by CYP71D189 and then to (+)‐lupanine by SDR1 (grey background).

To find the second oxidase, we first tested CYP76E36 and CYP71A168 in *N. benthamiana* by co‐expressing them individually with CYP71D189 and then feeding (−)‐sparteine. Co‐expression of neither cytochrome P450 changed the metabolite profile compared to a negative GFP control (Figure S2). Thus, we modified our selection strategy to find additional candidate genes. We focused on short‐chain dehydrogenase/reductases (SDRs) given that several SDRs have been shown to aid cytochrome P450s in the oxidation of fully reduced carbons to carbonyl compounds in plant specialized metabolism (Miettinen *et al*., 2014; Paddon *et al*., 2013). Only one SDR candidate (LOC109337773) was found when applying the following three criteria to all NLL transcripts in the aforementioned transcriptomics datasets: (i) high expression in leaves (highly active biosynthetic organs), (ii) higher expression in leaves of a high‐QA variety compared to a low‐QA variety and (iii) absence of closely related homologues in non‐QA‐containing plants. We named the SDR candidate SDR1 (see expression pattern in Table S1). Gratifyingly, co‐expression of SDR1 and CYP71D189 in *N. benthamiana* leaves led to decreased levels of the didehydrosparteinium ion as well as the appearance of (+)‐lupanine upon infiltration with (−)‐sparteine (Figure 3a,b). We propose that SDR1 acted on the immediate product of CYP71D189, 2‐hydroxysparteine (Figure 3c).

**Figure 4:**
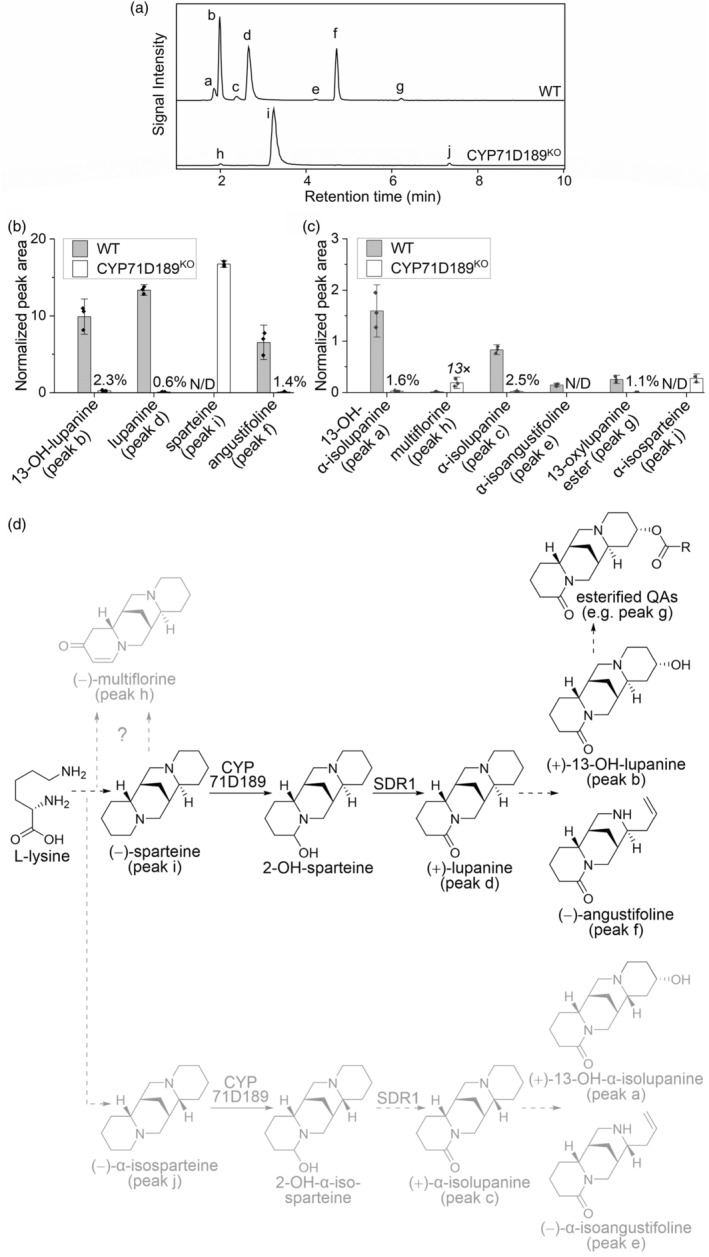
Production of (−)‐sparteine in NLL by genetic inactivation of CYP71D189. (a) LC–MS analysis of extracts of wild‐type (WT) or CYP71D189^KO^ NLL seeds. Traces are representative extracted ion chromatograms at the combined *m/z* ratios of 235.18 ± 0.01 (angustifoline), 235.22 ± 0.01 (sparteine), 247.18 ± 0.01 (multiflorine), 249.20 ± 0.01 (lupanine), 265.19 ± 0.01 (13‐hydroxylupanine), and 429.24 ± 0.01 (13‐[2‐OH‐3‐(4‐OH‐phenyl)propanoyl]oxylupanine) ([M + H]^+^ in all cases). Peak labels correspond to compounds shown in panel d and quantified in panels b and c. (b) Distribution and relative abundance of the four major QAs detected in WT vs. CYP71D189^KO^ seeds (*n* = 3). Data points are peak areas normalized by internal standard (caffeine) and dry seed weight. Bar charts represent mean values ±1.5 SD. Percentages represent the residual content of individual QAs in CYP71D189^KO^ vs. WT seeds. (c) Distribution and relative abundance of six minor QAs detected in WT vs. CYP71D189^KO^ seeds (*n* = 3) in the style of panel b. As multiflorine was more abundant in CYP71D189^KO^ seeds, the difference compared to WT is shown as a fold change. (d) Proposed QA biosynthesis pathway in NLL. The major QA branch is drawn in black and the minor branches are drawn in grey. Full arrows indicate steps catalysed by known enzymes; dashed arrows indicate putative steps.

Motivated by the prospect of generating a (−)‐sparteine‐accumulating NLL line, we isolated a CYP71D189 knockout from our recently constructed, non‐GMO NLL mutant library (Mancinotti *et al*., 2023) (Figure S3). The basis for the library was the bitter cultivar Oskar, whose seeds accumulate high levels of (+)‐lupanine, (+)‐13‐hydroxylupanine, and (−)‐angustifoline. The homozygous knockout mutants (CYP71D189^KO^) presented much reduced amounts of these major QAs in seed extracts, with only 0.6%, 2.3%, and 1.4% left, respectively. In their place, knockout seeds accumulated large amounts of (−)‐sparteine, which could not be detected in extracts of wild‐type seeds (Figure 4a,b). The levels of (−)‐sparteine in the extracts accounted for 1.2% of the weight of the mature seeds. For completeness, we also analysed five minor QAs also present in Oskar seeds: (−)‐multiflorine, (+)‐α‐isolupanine, (+)‐13‐hydroxy‐α‐isolupanine, (−)‐α‐isoangustifoline, and an ester of (+)‐13‐hydroxylupanine. With the exception of (−)‐multiflorine, all the minor QAs were strongly decreased in mutant seeds, with residual amounts ranging from undetectable [(−)‐α‐isoangustifoline] to 2.4% [(+)‐α‐isolupanine] (Figure 4a,c). (−)‐multiflorine, however, increased 13× starting from a trace amount in wild‐type seeds (Figure 4c). In addition, we detected the appearance of a minor amount of (−)‐α‐isosparteine in mutant seeds (Figure 4a,c). Based on the overall results described above, we conclude that CYP71D189 and SDR1 act in the major QA route represented by (−)‐sparteine as well as in the minor one represented by (−)‐α‐isosparteine (Figure 4d). Furthermore, we suggest that (−)‐multiflorine is produced from either (−)‐sparteine or the respective di‐iminium cation precursor (Figure 4d). Our results confirm the intermediacy of (−)‐sparteine in relation to (+)‐lupanine and its derivatives, which has not been clear in recent depictions of the general QA pathway (Cely‐Veloza *et al*., 2023; Ramírez‐Betancourt *et al*., 2021).

**Figure 5:**
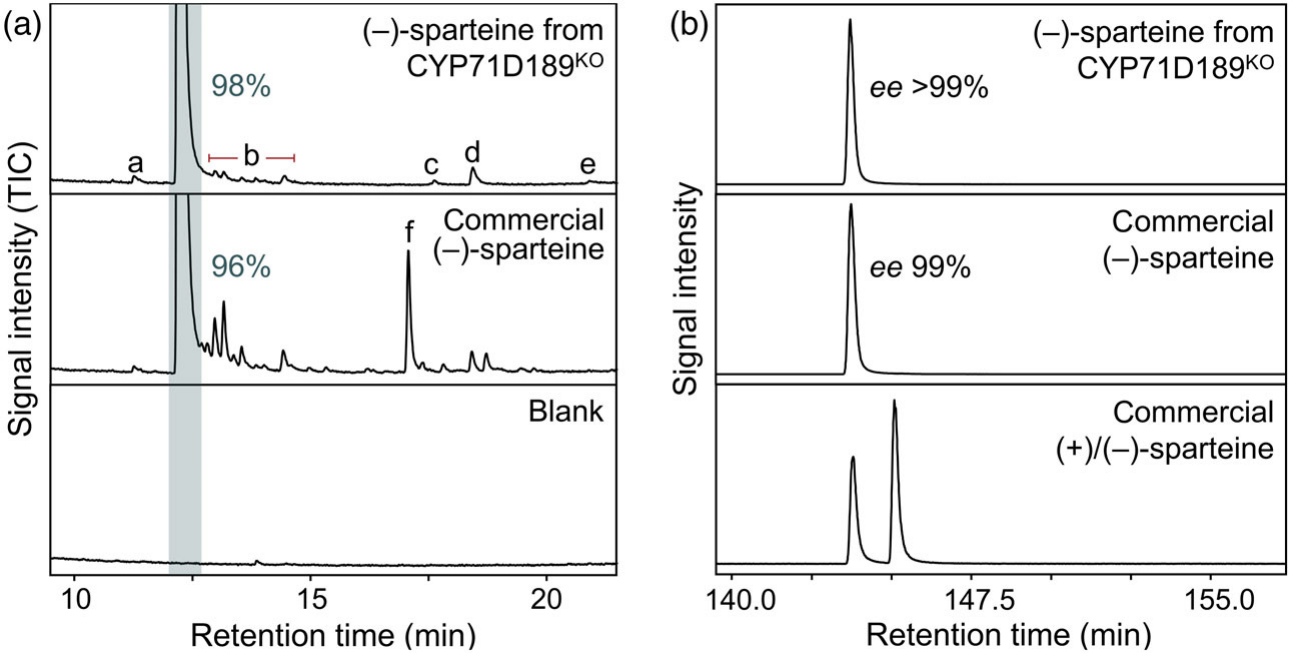
Comparison between commercial (−)‐sparteine and (−)‐sparteine isolated from CYP71D189^KO^ seeds. (a) GC–MS analysis. The main contaminants of the isolated (−)‐sparteine (98% pure) were α‐isosparteine (peak a), various didehydrosparteine species (peaks b), α‐isolupanine (peak c), lupanine (peak d), and multiflorine (peak e). In the commercial (−)‐sparteine (96% pure), the main contaminant was an unknown oxosparteine species (peak f), which could have arisen from air oxidation due to prolonged storage. GC–MS traces are total ion chromatograms (TICs) and are zoomed around the baseline to show minor peaks. The sparteine peak is highlighted by the grey band. (b) Chiral GC–MS analysis. The enantiomeric excess (*ee*) of (−)‐sparteine from CYP71D189^KO^ is comparable to or higher than that of the commercial product. Chromatograms are representative extracted ion chromatograms at *m/z* 137 ± 0.5 (base peak of sparteine).

We then isolated (−)‐sparteine from the relatively small M_5_ seed batch (Figure S3) using acid–base extraction. Compared to a crude methanolic extract, this simple procedure enabled an enrichment from 61% of total LC–MS peak area to 96% (Figure S4a), albeit at a yield reduction from 0.8% to 0.2% (dry seed weight). In addition, we subjected the purified (−)‐sparteine to chiral GC–MS analysis, which revealed an enantiomeric excess (*ee*) higher than that of our commercial standard (99% compared to 97%) (Figure S4b). Finally, we isolated (−)‐sparteine from the larger M_6_ seed batch (Figure S3), including crystallization, as bisulphate salt. In this case, the average yield was 0.3% (dry seed weight, assuming 100% purity of the isolated salt), with a purity of 98% as analysed by non‐chiral GC–MS (Figure 5a) and an *ee* of >99% as analysed by chiral GC–MS (Figure 5b).

Disruption of key biosynthetic genes is an effective strategy for introducing metabolic changes in an organism, and many crop plants have been engineered for superior content of desirable compounds using this approach (Dong *et al*., 2020; Jiang *et al*., 2017; Kaur *et al*., 2020; Li *et al*., 2022; Morineau *et al*., 2017; Zeng *et al*., 2021). With one notable exception (Li *et al*., 2022), our CYP71D189^KO^ NLL stands out in that our target metabolite (−)‐sparteine was previously absent from the original plant. Our work also demonstrates the potential of FIND‐IT technology (Knudsen *et al*., 2022) as an alternative to CRISPR‐Cas9 for metabolic engineering of plants for which transformation is unprecedented or technically challenging, such as for lupins. Since our NLL mutant library was created using EMS mutagenesis, CYP71D189^KO^ plants are also exempt from GMO regulations worldwide and do not require regulatory approval to be grown in the field. In our small‐scale experiments, we did not observe any visible phenotypes or apparent yield penalties compared to wild‐type plants. Furthermore, mature (dry) seeds can be ideal sites for long‐term storage, as they may offer an extra level of protection against air oxidation. We conclude that our NLL CYP71D189^KO^ plants are a valuable source of (−)‐sparteine for academia and industry.

## Experimental procedures

### Analytical methods

#### LC–MS

LC–MS analyses were carried out on a Thermo Fisher Dionex 3000 RS HPLC/UPLC system interfaced to a Bruker compact QqTOF mass spectrometer through an ESI source. Two different LC methods were used as described below. ESI mass spectra (*m/z* 50–1000) were acquired in positive ionization mode with automatic MS^2^ acquisition using the following parameters: capillary voltage 4500 V; end plate offset −500 V; source temperature 250 °C; desolvation gas flow 8.0 L/min; and nebulizer pressure 2.5 bar. N_2_ was used as desolvation, nebulizer and collision cell gas. Sparteine and lupanine were identified by comparison with known standards. (+)‐ and (−)‐sparteine were purchased from Sigma‐Aldrich (St. Louis, MO, USA). (+)‐ and *rac*‐lupanine were purchased from Innosil (Poznan, Poland). The identity of the other QAs was inferred from their predicted molecular formula and their mass spectral pattern, as shown by Otterbach *et al*. (2019).

#### LC method 1 (screen of oxidase candidates in *N. benthamiana* fed with (−)‐sparteine)

Analytes were separated at 40 °C on a Kinetex XB‐C18 column (100 × 2.1 mm, 1.7 μm, 100 Å, Phenomenex). Mobile phases A and B consist of 0.05% formic acid in water and 0.05% formic acid in acetonitrile, respectively. Analytes were eluted using the following gradient at a constant flow rate of 0.3 mL/min: 0–1 min, 2% B (constant); 1–16 min, 2–25% B (linear); 16–24 min, 25–65% B (linear); 24–26 min, 65–100% B (linear); 26–27 min, 100% B (constant); 27–27.5 min, 100–2% B (linear); and 27.5–33 min, 2% B (constant).

#### LC method 2 (test of CYP71D189 with or without SDR1 in *N. benthamiana* fed with (−)‐sparteine; QA quantification in seeds of the CYP71D189^KO^ NLL mutant)

Analytes were separated at 40 °C on a Luna C18(2) column (150 × 2 mm, 3 μm, 100 Å, Phenomenex). Mobile phases A and B consisted of, respectively, 0.05% formic acid in water and 0.05% formic acid in acetonitrile. Analytes were eluted using the following gradient at a constant flow rate of 0.3 mL/min: 0–0.5 min, 2% B (constant); 0.5–2.375 min, 2–6% B (linear); 2.375–7 min, 6–25% B (linear); 7–13 min, 25–100% B (linear); 13–14 min, 100% B (constant); 14–14.5 min, 100–2% B (linear); and 14.5–20 min, 2% B (constant).

#### LC method 3 (chiral separation of lupanine enantiomers)

Analytes were separated at 40 °C on a Lux AMP column (150 × 3 mm, 3 μm, Phenomenex). Mobile phases A and B consisted of 5 mm NH_4_HCO_3_ in water (adjusted to pH 11 with 25% NH_3(aq)_) and 2‐propanol, respectively. Analytes were eluted using the following gradient at a constant flow rate of 0.3 mL/min: 0–5 min, 15% B (constant); 5–30 min, 15–50% B (linear); 30–35 min, 50% B (constant); 35–36 min, 50–15% B (linear); 36–43 min, 15% B (constant).

#### GC–MS

Non‐chiral GC–MS analysis was carried out on a Shimadzu GCMS‐QP2010 Plus single quadrupole gas chromatograph‐mass spectrometer equipped with a Shimadzu AOC‐5000 autosampler. Analytes were separated on an Agilent J&W HP‐5 ms Ultra Inert capillary column (30 m × 0.25 mm, 0.25 μm) using He as carrier gas. Ethyl acetate extracts were injected in splitless mode (1 μL) at an inlet temperature of 250 °C. Analytes were separated using the following column temperature program at a constant carrier gas linear velocity of 33.7 cm/s: initial 80 °C, hold for 3 min; ramp to 150 °C at 30 °C/min; ramp to 300 °C at 6 °C/min; hold for 10 min. The separated analytes were ionized using an electron impact ion source at 250 °C. MS spectra were acquired in scan mode (m/z 30–600) at an energy of 70 eV. The purity of the extracted sparteine was estimated using the %area normalization method. In the calculation of the total peak area, we included all chromatographic peaks visible in the total ion chromatogram of the extract but absent in that of a blank.

#### Chiral GC–MS analysis

Chiral GC–MS was carried out on a Shimadzu Nexis GC‐2030 gas chromatograph equipped with a Shimadzu AOC‐6000 autosampler and coupled to a Shimadzu GCMS‐QP2020 NX single quadrupole mass spectrometer. Analytes were separated on an Agilent J&W CycloSil‐B capillary column (30 m × 0.25 mm × 0.25 μm) using He as carrier gas. Ethyl acetate extracts were injected in splitless mode (1 μL) at an inlet temperature of 250 °C. For the chiral analysis of (−)‐sparteine extracted from the M_5_ generation of CYP71D189^KO^ seeds, analytes were separated using the following column temperature program at a constant carrier gas pressure of 51.0 kPa: initial 80 °C; hold for 3 min; ramp to 125 °C at 30 °C/min; ramp to 240 °C at 2 °C/min. For the chiral analysis of (−)‐sparteine bisulphate isolated from the M_6_ generation of CYP71D189^KO^ seeds, analytes were separated using the following column temperature program at a constant carrier gas linear velocity of 33.7 cm/s: initial 80 °C; hold for 3 min; ramp to 125 °C at 30 °C/min; hold for 120 min; ramp to 240 °C at 2 °C/min. The separated analytes were ionized using an electron impact ion source at 250 °C. MS spectra were acquired in scan mode (m/z 10–300) at an energy of 70 eV. (+)‐ and (−)‐sparteine were identified by comparison with commercial standards from Sigma‐Aldrich (St. Louis, MO, USA). The enantiomeric excess of (−)‐sparteine was calculated from the extracted ion chromatogram of *m/z* 137 ± 0.5 (base peak of sparteine).

### Determination of the enantiomeric purity of lupanine in NLL

Seeds and dried leaves of NLL cv. Oskar were ground to a fine powder. ~20 mg of seed flour or ~20 mg of dried leaf powder were extracted in 1 mL of extractant (60% methanol, 0.06% formic acid) by shaking vigorously for 1 h. The extracts were briefly centrifuged to remove solid residues, diluted 15× with ultrapure water, and filtered through a 0.22‐μm filter. The filtered extracts were analysed by LC–MS as described above under Analytical methods (LC method 3).

### Selection of gene candidates

For gene candidate selection, we used an NLL transcriptomics dataset including eight different organs of the bitter cultivar Oskar (NCBI BioProject PRJNA386115) and five different organs of the sweet cultivar Tanjil (NCBI BioProject PRJNA248164). This includes biosynthetic as well as non‐biosynthetic organs for both cultivars. The RNA‐Seq reads from both cultivars were mapped onto the Oskar transcriptome (Yang *et al*., 2017) using Kallisto (Bray *et al*., 2016) v0.43.0, and gene expression was quantified in transcripts per million (TPM).

The selection of candidate genes for the oxidation of sparteine was done by co‐expression analysis using *LDC* as bait. We first reduced our dataset by removing all those transcripts with low expression (<10 TPM) in young Oskar leaves. Then, we calculated Pearson's correlation coefficients (PCCs) between *LDC* and all other transcripts. The transcripts with PCC higher than 0.9 were subjected to BLASTX against green plant protein sequences from NCBI (taxid: 33090). We distinguished between general and specific transcripts based on the %ID of the first 30 BLASTX results. Specifically, we only retained specific transcripts defined as those with 10 or more BLASTX results with %ID < 70%. We searched among the annotations of the remaining transcripts for keywords indicating oxidative enzymes, thus narrowing down to three hits. The three transcripts were predicted to encode cytochrome P450s, specifically a *CYP76E*, a *CYP71A*, and a *CYP71D* (Table S1).

To obtain additional candidates for the oxidation of 2‐hydroxysparteine, we expanded our scope to include genes that were less strongly co‐expressed with *LDC* yet had higher expression in Oskar vs. Tanjil. Hence, we filtered our dataset by removing all those transcripts with low expression (<10 TPM) in young Oskar leaves and with higher expression in leaves of Tanjil vs. Oskar (TPM ratio >1). The remaining transcripts were subjected to BLASTX and selection of specific transcripts based on %ID as described above. Finally, we searched for the keyword “dehydrogenase” among the annotations of the remaining transcripts and obtained only two hits. One of them appeared to encode a fragment of subunit CRR3 of the NAD(P)H dehydrogenase complex and was therefore deemed unlikely to be involved in the biosynthesis of QAs. The other transcript was predicted to encode an SDR specifically annotated as (−)‐isopiperitenol/(−)‐carveol dehydrogenase (Table S1).

### Transient expression of biosynthetic gene candidates in Nicotiana benthamiana

Total RNA was extracted from young leaves of narrow‐leafed lupin (NLL) cv. Oskar using the Spectrum Plant Total RNA Kit from Sigma‐Aldrich (St. Louis, MO, USA), and cDNA was synthesized using the iScript cDNA Synthesis Kit from Bio‐Rad (Hercules, CA, USA). Full‐length coding sequences of *CYP71A168*, *CYP71D189*, *CYP76E36*, and *SDR1* (see Gene sequences  section in the Supporting information S1) were amplified from the NLL leaf cDNA using the primers shown in Table S2. The coding sequence of mGFP5 (GFP) was amplified from the plasmid pCAMBIA1302 using the primers shown in Table S2. The genes were cloned into the plant expression vector pEAQ‐USER (Luo *et al*., 2016) by USER cloning (Nour‐Eldin *et al*., 2010) and transformed into *E. coli* strain Top10. Positive clones were verified by Sanger sequencing and transformed into *Agrobacterium tumefaciens* strain AGL‐1. For agroinfiltration, *Agrobacterium* strains were grown in YEP liquid medium supplemented with 50 μg/mL kanamycin, 25 μg/mL rifampicin and 50 μg/mL carbenicillin at 28 °C and 220 rpm until OD_600_ ≈ 3–4 in conical flasks. Bacterial pellets were harvested by centrifugation and resuspended in ultrapure water to OD_600_ = 1. Different strains were mixed in equal portions to obtain the desired combination of genes for co‐expression in the leaves of *N. benthamiana*. A strain expressing GFP was used as a negative control. After 1–3 h of incubation at room temperature, the mixtures were infiltrated into the abaxial side of young leaves of 4‐week‐old *N. benthamiana* plants using a needle‐less 3‐mL syringe. Infiltrated plants were allowed to recover overnight in the dark before being taken back into the greenhouse. To feed (−)‐sparteine and (+)‐lupanine, a 50‐ppm solution was prepared in buffer (10 mm MgCl_2_, 10 mm Na‐MES buffer pH 5.6), and the solution was infiltrated into the abaxial side of previously agroinfiltrated leaves at 4–5 days post agroinfiltration. Care was taken to infuse the solutions into the entirety of the previously agroinfiltrated area, which appeared discoloured. The fed plants were grown for an additional 4–5 days. Two leaf discs of 1‐cm diameter were harvested from the agroinfiltrated (discoloured) portions of each leaf at 8–10 days post agroinfiltration. The leaf discs were either quickly frozen in liquid nitrogen (screening of the three CYP candidates in leaves fed with (−)‐sparteine) or dried overnight in an oven at 40 °C (all other experiments). The frozen or dried leaf discs were pulverized using steel beads and a TissueLyzer bead beater from Qiagen (Hilden, Germany). After removal of the beads, the leaf powder was extracted with 250 μL of extractant (60% methanol, 0.06% formic acid, and 5 ppm caffeine in water) for 3 h at 1200 rpm. The extracts were briefly centrifuged to remove leaf debris, diluted 5× with ultrapure water, and filtered through a 0.22‐μm filter. The filtered extracts were analysed by LC–MS as described above under Analytical methods (LC method 1 and 2).

### Isolation and initial characterization of CYP71D189^KO^ NLL plants

The NLL mutant library constructed previously (Mancinotti *et al*., 2023) was screened essentially as described in (Knudsen *et al*., 2022) to identify a mutant seed harbouring the specific G to A nucleotide change at position 647 in the coding region of *CYP71D189* (corresponding to W216 Stop; see the Gene sequences  section in the Supporting information S1). For the TaqMan assays, we used the following primers: a target‐specific forward primer (CYP71D189_TaqMan_FW), a target‐specific reverse primer (CYP71D189_TaqMan_RV), a WT‐specific probe containing a HEX fluorophore and a BHQ1 quencher (CYP71D189WT_TaqMan_HEX), and a mutant‐specific probe containing a FAM fluorophore and a BHQ1 quencher (CYP71D189KO_TaqMan_FAM) (Table S2). One heterozygous mutant seed was retrieved. The M_2_ heterozygous plant that grew from the seed was allowed to self‐pollinate. The resulting M_3_ seeds were sown in 16 cm‐wide, 20 cm‐deep pots filled with commercial peat‐based potting soil and grown in a growth cabinet with a light/dark photoperiod of 16/8 h, a day/night temperature of 21/18 °C, and a relative humidity of 60%. Young leaves from the M_3_ plants were quickly frozen in liquid nitrogen and pulverized with the help of steel beads and a TissueLyzer bead beater from Qiagen (Hilden, Germany). Genomic DNA was extracted from the leaves using the E.Z.N.A.® Plant DNA DS Kit from Omega Bio‐tek (Norcross, GA, USA), and genotyping was carried out by Sanger sequencing of a PCR fragment spanning the entire gene between its start and stop codons using primers CYP71D189_pEAQ_FW and CYP71D189_pEAQ_RV (Table S2). All the M_3_ WT plants and homozygous CYP71D189^KO^ plants were allowed to self‐pollinate. For QA analysis, three mature, dry M_4_ seeds from either three WT plants or three homozygous CYP71D189^KO^ plants were pulverized using a steel ball with the help of a TissueLyzer bead beater from Qiagen (Hilden, Germany). QAs were extracted from ~20 mg of seed flour in 1 mL of extractant (60% methanol, 0.06% formic acid, and 15 ppm caffeine in water) for 3 h at 1200 rpm. The extracts were briefly centrifuged to remove solid residues, diluted 15× with ultrapure water, and filtered through a 0.22‐μm filter. The filtered extracts were analysed by LC–MS as described above under Analytical methods (LC method 2).

### Purification and chiral analysis of (−)‐sparteine from M_5_ CYP71D189^KO^ seeds

Fifty M_4_ seeds from one M_3_ homozygous CYP71D189^KO^ plant were grown in the field at Nørre Aaby, Fyn, Denmark. The seeds were hand sown in two separate rows 25 cm apart during April of 2022. 44 plants reached maturity and were harvested in late August 2022, yielding 438 g of M_5_ seeds. 12 seeds were pooled and pulverized using a steel ball and a TissueLyzer bead beater from Qiagen (Hilden, Germany).

For the methanolic extraction, ~15 mg of seed flour were extracted in 1 mL of extractant (60% methanol, 0.06% formic acid, and 15 ppm caffeine in water) for 3 h at 1200 rpm. The extracts were cleared by centrifugation and diluted 15× with ultrapure water. After filtration through a 0.22‐μm filter, the extracts were analysed by LC–MS as described above under Analytical methods (LC method 2). Sparteine was quantified using a calibration curve, with caffeine used as an internal standard.

For the acid–base extraction, 250 mg of seed flour were extracted in 2 mL of 1 m HCl_(aq)_ by shaking at 400 rpm for 1 h at room temperature. Debris was removed by centrifugation, and the cleared acidic extract was defatted with n‐hexane (5× 1‐mL portions). The defatted extract was basified with 1.5 mL of 2 m NaOH_(aq)_ and extracted with dichloromethane (5× 1‐mL portions). The organic extract was dried over anhydrous Na_2_SO_4_ and evaporated under reduced pressure. The residual oil was redissolved in 500 μL of ethyl acetate. For determination of the enantiomeric excess, the extract was diluted 5× in ethyl acetate before chiral GC–MS analysis as described under Analytical methods.

For the determination of the purity of (−)‐sparteine upon acid–base extraction, 15 μL of the ethyl acetate extract were evaporated by gentle heating, and the residue was redissolved in 50 μL of 60% aqueous methanol containing 0.06% formic acid. The sample was then diluted 15× with ultrapure water, filtered through a 0.22‐μm filter, and analysed by LC–MS as described above under Analytical methods (LC method 2). The purity of the extracted sparteine was estimated using the %area normalization method. All chromatographic peaks visible in the total ion chromatogram of the extract but absent in that of a blank extraction sample were included in the calculation of the total peak area.

### Isolation and chiral analysis of (−)‐sparteine from M_6_ CYP71D189^KO^ seeds

300 g of M_5_ seeds derived from forty‐four M_4_ CYP71D189^KO^ homozygous plants were grown in the field in Christchurch, New Zealand. The seeds were machine‐sown in a single plot in November 2022. 3 kg of M_6_ seeds were harvested in April 2023. The seeds were pooled, and a 100‐g portion was pulverized using a steel ball and a TissueLyzer bead beater from Qiagen (Hilden, Germany).

10 g of seed flour were extracted in 200 mL of 0.5 m H_2_SO_4(aq)_ by stirring at 600 rpm for 18 h at room temperature. Large debris was removed by centrifugation. The yellow oil layer that formed above the extract was discarded by pipetting. The extract was basified with 35 mL of 10 M NaOH_(aq)_ and centrifuged again to remove the precipitate that formed upon addition of the base. The basified extract was extracted with diethyl ether (5 × 50‐mL portions). The organic extract was dried over anhydrous Na_2_SO_4_ and evaporated under reduced pressure. The residual oil was redissolved in 2 mL of isopropanol. 2 mL of 0.5 m H_2_SO_4_ in isopropanol were then added to precipitate (−)‐sparteine as its bisulphate salt. The mixture was kept for 12 h at 4 °C, and the resulting crystals were collected by vacuum filtration, washed with ice‐cold isopropanol, and dried over the filter. (−)‐Sparteine bisulphate (C_15_H_26_N_2_∙2H_2_SO_4_; 55–71 mg, 0.30–0.39% of seed dry weight) appeared as a white, crystalline powder which decomposed upon heating to 262–264 °C (m.p. lit (Firth *et al*., 2018). 247–250 °C, decomp.).

To determine the enantiomeric excess and the purity of the isolated (−)‐sparteine bisulphate by GC–MS, samples were prepared by dissolving 3 mg of (−)‐sparteine bisulphate or commercial (−)‐sparteine in 1 mL of 5 mm H_2_SO_4(aq)_. 500 μL of the sparteine solutions were basified with 50 μL 10 m NaOH_(aq)_ and extracted with 2 mL ethyl acetate. The organic extract was dried over anhydrous Na_2_SO_4_, diluted 5× in ethyl acetate, and analysed as described above under Analytical methods.

## Accession numbers

The sequences of CYP71D189^WT^, CYP71D189^KO^, CYP76E36, CYP71A168, and SDR1 are available in the Supplementary material.

## Conflict of interest

The authors declare being the inventors of a patent application filed by the University of Copenhagen on the subject matter of this article (PCT/EP2023/087645).

## Supporting information


**Figure S1** Occurrence of enantiomerically pure (+)‐lupanine in NLL.
**Figure S2** CYP71A168 and CYP76E36 cannot oxidize 2‐hydroxysparteine to lupanine.
**Figure S3** Propagation of the CYP71D189^KO^ mutant.
**Figure S4** Small‐scale purification of (−)‐sparteine from the M5 generation of CYP71D189^KO^ mutant seeds.
**Table S1** Candidate genes for the oxidation of sparteine to lupanine in NLL.
**Table S2** List of DNA oligos used in this study.

## Data Availability

We used publicly available lupin RNA‐Seq datasets from the National Center for Biotechnology Information (BioProject PRJNA386115 and PRJNA248164). The sequences of the three CYPs and of SDR1 are included in the Supplementary material.
