# Supplementary material for: Metabolic engineering of narrow‐leafed lupin for the production of enantiomerically pure (−)‐sparteine
Source: Plant Biotechnol J. 2024 Nov 22;23(2):467–76. doi: 10.1111/pbi.14509 (PMC11772310; doi:10.1111/pbi.14509)
Supplement: Supplementary file 1 — Figure S1 Occurrence of enantiomerically pure (+)‐lupanine in NLL. Figure S2 CYP71A168 and CYP76E36 cannot oxidize 2‐hydroxysparteine to lupanine. Figure S3 Propagation of the CYP71D189KO mutant. Figure S4 Small‐scale purification of (−)‐sparteine from the M5 generation of CYP71D189KO mutant seeds. Table S1 Candidate genes for the oxidation of sparteine to lupanine in NLL. Table S2 List of DNA oligos used in this study. [file PBI-23-467-s001.docx]

Supporting Information

Metabolic engineering of narrow-leafed lupin for the production of enantiomerically pure (−)-sparteine

Davide Mancinotti^1^, Ting Yang^1^, and Fernando Geu-Flores^1,^*

^1^Section for Plant Biochemistry and Copenhagen Plant Science Centre, Department of Plant and Environmental Sciences, University of Copenhagen, Frederiksberg, Denmark

*Correspondence to: [feg@plen.ku.dk](mailto:feg@plen.ku.dk)

**TABLE OF CONTENTS**

[Supplementary Figures 1](#_TOC_250002)

**Fig. S1.** Occurrence of enantiomerically pure (+)-lupanine in narrow-leafed lupin 1

**Fig. S2.** CYP71A168 and CYP76E36 cannot oxidize 2-hydroxysparteine to lupanine 2

**Fig. S3.** Propagation of the CYP71D189^KO^ mutant 3

**Fig. S4.** Small-scale purification of (–)-sparteine from the M5 generation of CYP71D189^KO^ mutant seeds 4

[Gene Sequences 5](#_TOC_250001)

CYP71D189^WT^ 5

CYP71D189^KO^ 6

SDR1 7

CYP76E36 7

CYP71A168 8

**Table S1.** Candidate genes for the oxidation of sparteine to lupanine in NLL **9**

Table S2. List of DNA oligos used in this study 10

[References 11](#_TOC_250000)

# Supplementary Figures


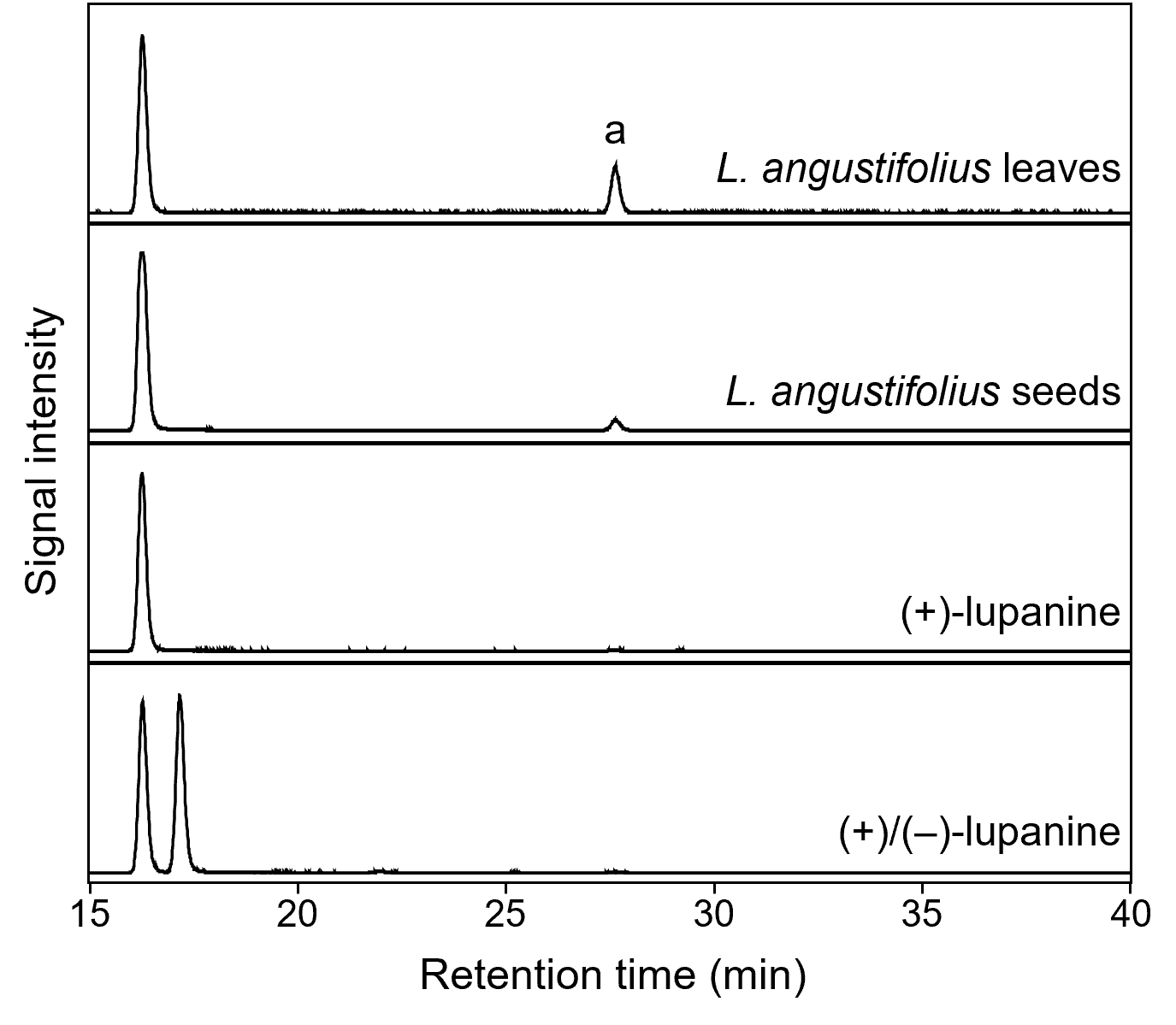


**Figure S1.** Enantiomeric purity of (+)-lupanine in narrow-leafed lupin (NLL, *Lupinus angustifolius*). Extracts of NLL seeds and leaves were analyzed by chiral LC-MS showing the enantiopurity of (+)-lupanine as well as small amounts of α- isolupanine (peak a). Traces are extracted ion chromatograms at *m/z* 249.20 ± 0.01 ([M+H]^+^ of lupanine). Traces belonging to standards of (+)-lupanine and racemic lupanine are also shown for comparison.


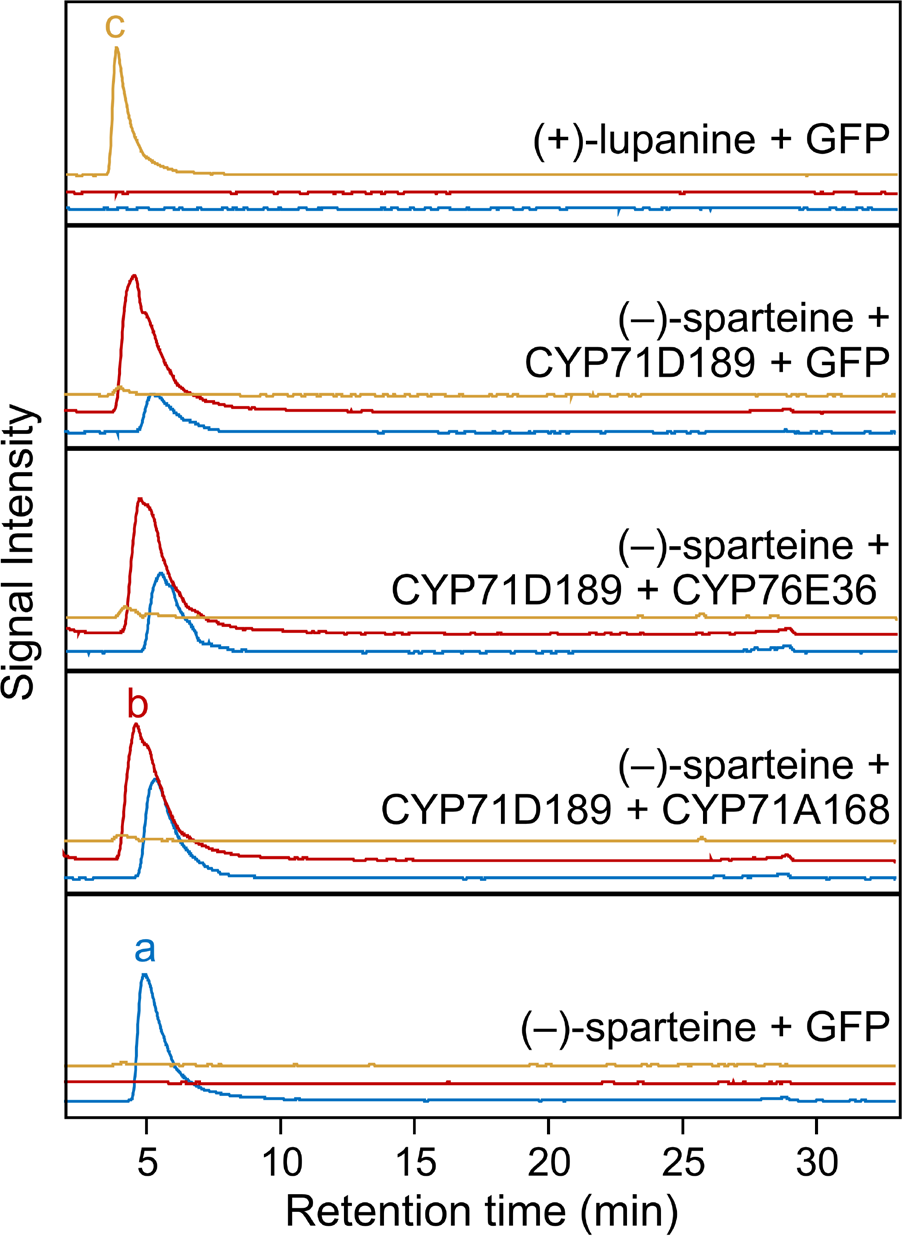


**Figure S2.** CYP71A168 and CYP76E36 cannot oxidize 2-hydroxysparteine to lupanine. LC-MS analysis of extracts of *N. benthamiana* leaves expressing CYP71D189 in separate combination with two other oxidase candidates at 10 dpi following feeding with (–)-sparteine at 5 days post infiltration. Peak a corresponds to (+)-lupanine, peak b to 1,2-didehydrosparteinium, and peak c to (–)-sparteine. None of the two oxidase candidates is capable of oxidizing (–)-sparteine further to (+)-lupanine. Leaves expressing GFP fed with (–)-sparteine or (+)-lupanine were included as controls. The traces are representative extracted ion chromatograms corresponding to sparteine ([M+H]^+^, m/z 235.22 ± 0.01, blue trace), 1,2-didehydrosparteinium (M^+^, m/z 233.20 ± 0.01, red trace), and lupanine ([M+H]^+^, m/z 249.20 ± 0.01, orange trace). The traces are slightly offset to aid visualization of the otherwise overlapping peaks.


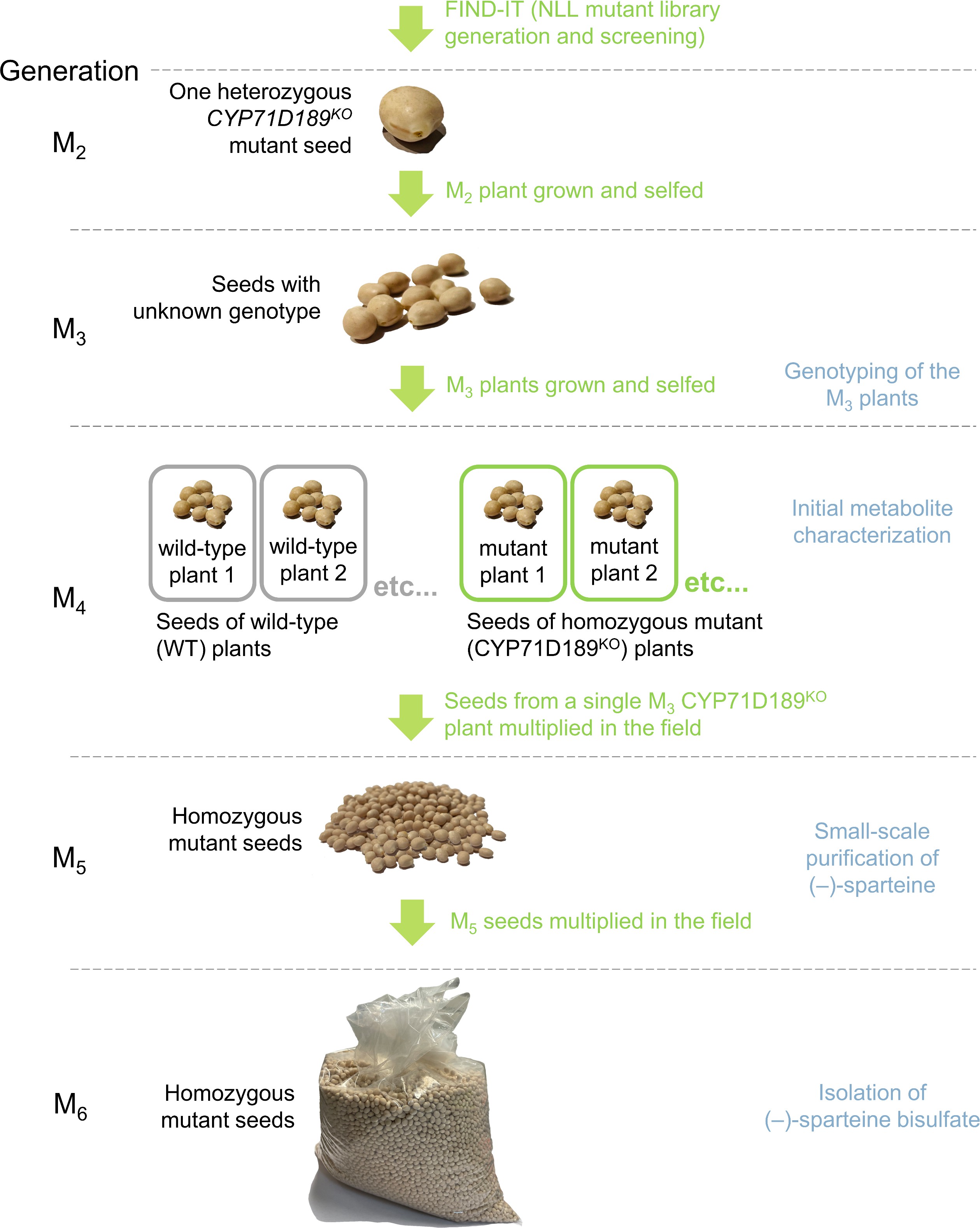


**Figure S3.** Propagation of the CYP71D189^KO^ mutant. We retrieved one M2 heterozygous *CYP71D189^KO^* mutant seed from our previously constructed NLL mutant library (Mancinotti *et al.*, 2023). We allowed the heterozygous M2 plant that grew from this seed to self-pollinate and grew a portion of the M3 seeds obtained from this plant under controlled conditions. We genotyped the resulting M3 plants (a mixture of wild type, heterozygous, and homozygous plants) and allowed wild-type plants and homozygous mutants (CYP71D189^KO^) to self-pollinate. We then grew the M4 seeds from a single M3 CYP71D189^KO^ plant in the field to give M5 seeds, and these were further multiplied to give the M6 generation.


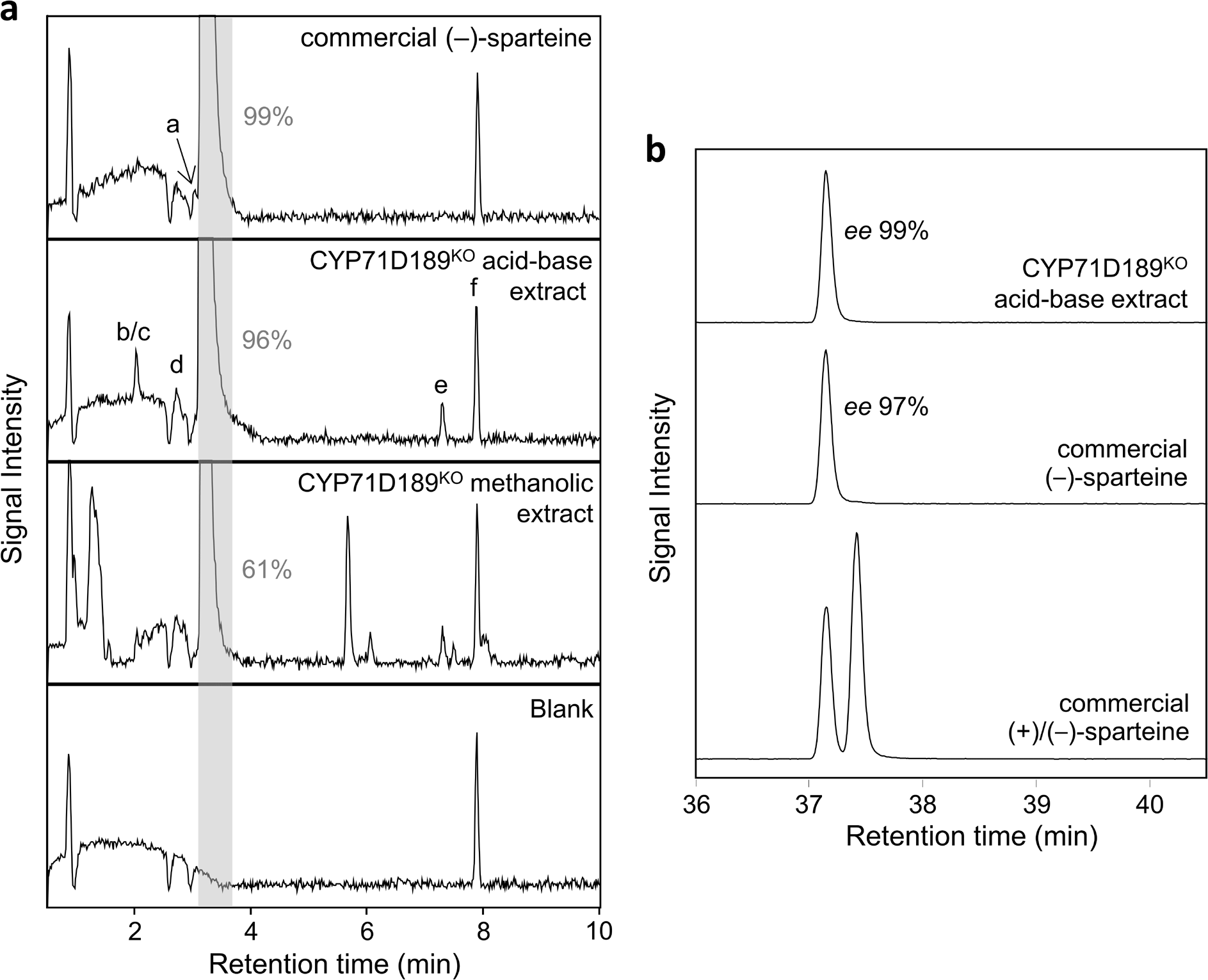


**Figure S4.** Small-scale purification of (–)-sparteine from the M5 generation of CYP71D189^KO^ mutant seeds. (a) LC-MS analysis of crude methanolic extracts and extracts purified by acid-base extraction. The acid-base extraction removed non-alkaloidal contaminants, leaving mostly sparteine (96% by LC-MS peak area) and a small amount (4%) of 13- hydroxylupanine (peak b), multiflorine (peak c), lupanine (peak d) and α-isosparteine (peak e) as main contaminants. In the commercial (–)-sparteine (99% pure by LC-MS peak area), the main contaminant was found to be an unknown oxosparteine species (peak a), which could have arisen from air oxidation upon prolonged storage. LC-MS traces are total ion chromatograms and are zoomed around the baseline to show minor peaks. The sparteine peak is highlighted by the grey band. The peak around 8 min corresponds to the internal standard (caffeine, peak f). (b) Chiral GC-MS analysis of acid-base extracts from the seeds of CYP71D189^KO^ plants compared to commercial products. The enantiomeric excess (*ee*) of (–)-sparteine from CYP71D189^KO^ is comparable or higher to that of the commercial product (99% compared to 97%). Chromatograms are representative extracted ion chromatograms at m/z 137 ± 0.5 (base peak of sparteine).

# Gene sequences

*Sequence of CYP71D189^WT^. Exons are highlighted in grey.*

ATGGAGCTTCAAAACCCTTTCACTATTTTGTTTACATCATTTCTCTTTCTATTCTTGTTACTAGAAATAGTTAAGAGATCC AGTTCAAAGAATTCCTATACAAACTTACCACCAGGGCCATGGAAACTACCCTTCATAGGTAACATACACCAATTTGTTG GGTCAATGCCCCATCACTCCATGAGAGATTTAGCAGCCAAGTATGGTCCTATAATGCACCTAAAACTAGGAGAAGTTT CCAACATCATAGTTAGTTCAGCAGAAATTGCTAGCCAGATTATGAAAACACATGATGCCAATTTTTCTTACAGGCCAGA GAGTCTTTTTGCCAAAATATTTTCTTATAATGCTTCAGACATTGAATTCTCTCAATATGGAGATTATTGGAGGCAACTAA GAAAGATATGCACCGTCGAGTTGCTATCAGCAAAACGTGTTCAATCATTCAGGTCATAATTATCTTTATGACTTTTCTTA ATATTATATAATTCGGATGTGAATAAATTATTAACTGATATCTTTATTATTATATCAAAATATGTATCTTCATAAGATTTG GTATATAGTTTACTATTGTCAGAATCTAAGATCCCACGTACATATATATATATTATCTATATGCTGGCCTCATAACTTCA AATTTTTATGTGTGTTGCTAAATATTCAGGTTCATAAGAGAAGAGGAAGTGTCAAAACTTGCTAAAATAATATGTACAA GTGAGGGGTCCATTGTGAATCTGTCTCCTATGATTTCCTCATTGTTGCATGGGATATCAGCACGATCAGCTTTTGGTAA AATAAATAAAAATCAGAAAATATTGATATCAGCAATTGAGGAAGGAATTTTTCTAGCAGGAGAACTATGGGTTAGTG AATTCTATCCCTCATTAACAGTGCTTCAAAAGTTGAGTAAGACAAAGGCTAAACTTGAAAAGTTGCACATAGAGGCTG ATAAAATATTGCAAGAGATTCTTGATGATCACAAAAATAAAAAAAGCAGTGAGAGTGAAGATCTAATAGATGTTCTTC TCAAGTTTCAAAATGACAAGGATTCTCAACCTCCCTTAACTGATGACAATATTAAAGCAATTGGCCAGGTTAGTACATT TTCTCTATGTATGTCTAACATTATTCTTTATGTGTAAAAATTAGGTAGAATTTATAACATTATTTATGATGAATGATTTTA ATCAATCTCCATGAAATGATTGGTGTCCTCGATTATGTAATAAAGATTCATGTTTGTATGGAAGAAAATGTTTATGGAT AAGGGATTATGAATCCATTTATTATAGGAGATGTTTGGTGGTGGTGGTGAAACAACATCAAGTACTGTTGTGTGGTGT TTGTCAGAAATGATAAAGAAACCAAAAGTGATGGAAGAAGCACAAGCTGAGGTAAGAAAAGTGTATGATAATAAAG GATATGTGGATGAGTCAGAGTTGCACCAATTGATATATTTGAAGGCAGTGATCAAAGAAACACTGAGGCTCCACCCAC CTGTCCCATTGTTAATGCCAAGAGAAAATAAAGATAGCAGCAAAATCAATGGATATGATATACCACCTAAGTGCAAGG TCTTAATAAATGCTTTTGCTATTGGAAGAGATCCTAAGTATTGGAATGAACCTGAAAGCTTTAATCCTGAGAGATTTCT GAATAGTTCAGTAGATTATAAGGGCACAGACTTTGAGTTTATACCATTTGGTGCTGGAAGGAGGATATGCCCTGGTAT TACATTTGCCATACCAAACATGGAGCTTCCACTTGCAACATTGCTTTACCATTTTGATTGGAAGCTTCCAAATGGAATG AAGAATGAAGAACTTGATATGGATGAATCATCTGGGTTGGCTATTAAAAGAAAAAATGATCTTTGCTTGATTCCAATT GTTACTCGTATGCCTTAA

*Sequence of CYP71D189^KO^. Exons are highlighted in gray. The nonsense mutation is highlighted in yellow.*

ATGGAGCTTCAAAACCCTTTCACTATTTTGTTTACATCATTTCTCTTTCTATTCTTGTTACTAGAAATAGTTAAGAGATCC AGTTCAAAGAATTCCTATACAAACTTACCACCAGGGCCATGGAAACTACCCTTCATAGGTAACATACACCAATTTGTTG GGTCAATGCCCCATCACTCCATGAGAGATTTAGCAGCCAAGTATGGTCCTATAATGCACCTAAAACTAGGAGAAGTTT CCAACATCATAGTTAGTTCAGCAGAAATTGCTAGCCAGATTATGAAAACACATGATGCCAATTTTTCTTACAGGCCAGA GAGTCTTTTTGCCAAAATATTTTCTTATAATGCTTCAGACATTGAATTCTCTCAATATGGAGATTATTGGAGGCAACTAA GAAAGATATGCACCGTCGAGTTGCTATCAGCAAAACGTGTTCAATCATTCAGGTCATAATTATCTTTATGACTTTTCTTA ATATTATATAATTCGGATGTGAATAAATTATTAACTGATATCTTTATTATTATATCAAAATATGTATCTTCATAAGATTTG GTATATAGTTTACTATTGTCAGAATCTAAGATCCCACGTACATATATATATATTATCTATATGCTGGCCTCATAACTTCA AATTTTTATGTGTGTTGCTAAATATTCAGGTTCATAAGAGAAGAGGAAGTGTCAAAACTTGCTAAAATAATATGTACAA GTGAGGGGTCCATTGTGAATCTGTCTCCTATGATTTCCTCATTGTTGCATGGGATATCAGCACGATCAGCTTTTGGTAA AATAAATAAAAATCAGAAAATATTGATATCAGCAATTGAGGAAGGAATTTTTCTAGCAGGAGAACTATAGGTTAGTGA ATTCTATCCCTCATTAACAGTGCTTCAAAAGTTGAGTAAGACAAAGGCTAAACTTGAAAAGTTGCACATAGAGGCTGA TAAAATATTGCAAGAGATTCTTGATGATCACAAAAATAAAAAAAGCAGTGAGAGTGAAGATCTAATAGATGTTCTTCT CAAGTTTCAAAATGACAAGGATTCTCAACCTCCCTTAACTGATGACAATATTAAAGCAATTGGCCAGGTTAGTACATTT TCTCTATGTATGTCTAACATTATTCTTTATGTGTAAAAATTAGGTAGAATTTATAACATTATTTATGATGAATGATTTTAA TCAATCTCCATGAAATGATTGGTGTCCTCGATTATGTAATAAAGATTCATGTTTGTATGGAAGAAAATGTTTATGGATA AGGGATTATGAATCCATTTATTATAGGAGATGTTTGGTGGTGGTGGTGAAACAACATCAAGTACTGTTGTGTGGTGTT TGTCAGAAATGATAAAGAAACCAAAAGTGATGGAAGAAGCACAAGCTGAGGTAAGAAAAGTGTATGATAATAAAGG ATATGTGGATGAGTCAGAGTTGCACCAATTGATATATTTGAAGGCAGTGATCAAAGAAACACTGAGGCTCCACCCACC TGTCCCATTGTTAATGCCAAGAGAAAATAAAGATAGCAGCAAAATCAATGGATATGATATACCACCTAAGTGCAAGGT CTTAATAAATGCTTTTGCTATTGGAAGAGATCCTAAGTATTGGAATGAACCTGAAAGCTTTAATCCTGAGAGATTTCTG AATAGTTCAGTAGATTATAAGGGCACAGACTTTGAGTTTATACCATTTGGTGCTGGAAGGAGGATATGCCCTGGTATT ACATTTGCCATACCAAACATGGAGCTTCCACTTGCAACATTGCTTTACCATTTTGATTGGAAGCTTCCAAATGGAATGA AGAATGAAGAACTTGATATGGATGAATCATCTGGGTTGGCTATTAAAAGAAAAAATGATCTTTGCTTGATTCCAATTG TTACTCGTATGCCTTAA

*Coding sequence of SDR1*

ATGGTAGAAACTACTTCCAACAACAGTGGTGCAAAGCTAGCAGGCAAAGTAGCCATCGTCACCGGAGGATCCGGTGG CATCGGCGAGGCAGCGGCGCATGCCTTTGCTGATCAAGGTGCGCGTGTGGTAATTGCAGACGTTCAAGACGATCTTG GCAATAAAGTTGCTGAGTCCATCGGAACCAACAAGTGTACCTACATCCACTGCAACGTAGCAGATGAAGAACAAGTCC AAAACCTAATTCAATCAACGGTCAACACTTTCGGACAAATCGACATCATGTTCAGCAACGCTGGCATCAGCAGTGAGT TAGAACAAACCATCATTGAACTTGACATATCCGAACTTAACCGCTTGTTGGCCGTAAACGCTAGTGGAATGGCGGCGT GTGTGAAACACGCGGCACGTGCCATGGTGGACAAGCACGTGAGAGGAAGCATAGTATGCACTGGAAGCATATATGG AAGTAACGGTGGATCTGATGGAACGGATTACGCCATGTCGAAGCATGCATTGTTGGGTTTGGTGCGTTCGGCGAGTA TACAACTAGCGGAGCATGGAATAAGGGTGAACTGCGTTTCACCAAATGCATTGGTAACTCCGATGACGTTGAAATATT CAGGGAGTGAAGAGAAGGTTTATGAGCTTTGTGCAAAAACCGCGAGGCTGAAAGGAGTGATACTCACTGCTAAACAT ATTGCTGATGCTGTGTTATTTCTTGCTTCTAATGATTCAGAGTTTATCACTGGCCATGATCTTGTTGTGGATGGTTCCTA TATTGCTCCATGA

*Coding sequence of CYP76E36*

ATGGATTATCTAACACTTTTTCTACTCATTTCCTTTGTTTGGACAAGCATTTATGTCCTATTCTCCAAATTAGGAATCAAA ACATCCAAATATGCCCCAGGTCCATACCCTTTACCTATCATAGGTAACATCTTTGAACTTGGAAAACTCCCACACCAAAC ACTTTCTAAGCTCTCTCAAACTTATGGACCTATAATGACCTTAAAGTTTGGTAGTGTTACTGCTATAGTTATTTCCTCTCC ACAAGTAGCCAAAGAAGCACTTCAAAAAAATGACCAAGTTTTCTCTTTTAGGCCAACCCCAGATACCCTTAGGGCACA TGACCATCATATATACTCAGTGGCATGGATGCAGCCTTCAGCTGATTGGAGGGCCCTTAGGAAAGCTTGCGCAATCAA AGTGTTCTCATCTCGAATGCTCGATTCGACGCAATTTTTACGACAAAAGAAGGTGCAAGAGTTGATGGATTATGTTAA GGAAAGTTGCAAGAAAGGTGAGGCTTTGGATATTGGCAAGGCAACTTTTAAGACTGTGCTTAATTCTATATCAAACAC TTTGTTCTCTATGGACTTGGCTCATTATACTTCTGATAAGTTTCAAGAGTTCAAGGACATTATTTGTGGGATCACTGAAG AAGCTGGAAAGCCTAACTATGTGGATTATTTTCCAATCCTTAGTTTTCTTGATCCACAAGGTGCCCATGGAAGAATGAA GGGTTATTTTGGAAAGTTGATTAAATTTTTTGATGATCTTATAGAAGAAAGGCTACAATTAAGAGCTACACAAAAGGA ATCCAAGGCTTGCAAAGATGTTCTAGATTCTGTGCTAGAACTCATGCTGGAAGACAATTCTCAAATTACTAGGCTCCAT GTTTCGCATTTGTTTGTGGATTTATTCGTGGCTGGAATAGATACCACATCAATCACAATAGAATGGGCAATGGCAGAG TTGCTACGTAATCCAGAAAAGCTAAAAAAAGTTAGAAAAGAACTTCAACAAGTTACAAGCAAAGGTGAACAACTTGA AGAAACACACATATCAAAGCTTCCTTTCTTAGAAGCAGTGATTAAAGAAACTTTTCGTTTGCATCCACCAGCAGCATTC TTAGTGCCACGCATGTCAGGAGATAATGTTGAACTATGTGGTTACATGGTACCTAAAAATGCACAAATTATGATTAAT GCATGGGCCATGGGAAGAGATTCAAGTGTTTGGGCCAACCCAAATGAATTTATCCCTGAAAGATTCTTGAATAATGAG ATTGATTTTAAAGGTCAATATTTTGAGCTTATTCCTTTTGGTGCTGGAAGAAGGATTTGTCCTGGTTTACCATTGGCTTC TAAGACTGTGCACACTGTCTTGGCCTCACTTTTATGTGGCTATGATTGGAAGCTTGTTGATGGAGGAGAGGGAGAGA ATATGGATATGTCTGAGGAATATGGGCTTACCTTACATAAGGCACAACCTCTCCTAGTTATTCCTATCAAAGCATAA

*Coding sequence of CYP71A168*

ATGCCTCTTCTATCTATTCAAGAGAATCTACCATATGTGCTAAATTCAACTTTCTACTTATCTGTACTATGTTTCTTTGGT GTCTTATTTGTCTTTAAGATCACTAGAAGAAGCAAAACCAATTCACCACCCTCCCCTCCAAAACTACCCTTTATTGGGAA TCTTCATCAACTAGGCACATTCCCACACCGTTCCTTACAATCCTTATCTTACAAATATGGCCCTATGATGATGATGAAAA TGGGACAAATTCAAACGCTAGTGATTTCATCTTCTGATGTGGCCAGAGAAATATTCAAAAGCCATGATGCTGTTTTCTC CAACCGACCCACGGTCACAGGCTCCGACATCTTCTTGTATGGGTCCAAAGATGTGGCCTTTGCTCCCTACGGCGATGA GTGGAGACAAAAAAGAAAGATTTTAGTTCTTGAGCTTCTAAGCATGAAAAGGGTGCAATCTTTTCAACCCATAAGAGA AGAAGAAGTTGGTGAAATGCTTCATGCTATACGTGATGCATGCAGAAAGTCATCAACGGTGAACCTGACTGAGATGC TGATTGCAGCCTCTAACAACTTAAATTCTAGATGTGTTTTCGGACAAAAGTATGATACTGAAGATGGCAGTCCCAGCTT CGGAGATCTAGGAAGGAAGATGTTGGTACAGTTCACAGCTTTCTGTGTTGGAGATTTCTGGCCTTCATTGAGTTGGAT TGATACACTTTCAGGCCAAATTCCAAAATTTATGGAAACTTTTACTTCGTTAGATATTTTCCTAGAAAGAGTTATTAAAG AACACAGGGCTAAGATGAAGAGTAGTGATGATCAATCCGATAAGAAAGACTTCGTGGATATACTTCTTCAACTTCAAG GAGAAGATAAGCTTGACTTTGAGCTCACCCAAGATATCCTCAAAGCACTAATAGTGAACTTGTTCATTGGAGGAAGTG ATACTTCATCAACAACAATGGAATGGGCTTTTGCAGAACTCATGAGGAATCCAAGGGTCCTGAAGAAAGCCCAAGAA GAGGTAAGAAGAGTTGTGGGGGACAAAAAAGTTGTAGATGCAAATGATACAAAACATATGAATTACTTGAAATGTGT AATCAAAGAAACTTTAAGATTACATCCACCAGCTCCTCTCTTGGTTCCTAGAGAAACAACTGCTACTGTTAATCTAAAA GGATATGACATTCCCTCCAAAACAAGGATACTTATAAATGGATTTGCAATCCAAAGGGACCCTGAAGTTTGGGACAAA GCTGATGAGTTTTACCCAGATAGATTCGAGAACAGTGAGGTTGACTTCAAAACACAAGACGTAGAATTTATAGCATTT GGCGGTGGAAGAAGGGGGTGCCCTGCAATTACATTTGCTGTTACCTTTACTACTTATGTGCTTGCTAATCTTCTATATT GGTTTGATTGGAAGCTTCCTGAAAACGTAGACGAGGTGGACATGAGTGAGAGATATGGAATTGTTGTCAACTTGAAA GTACCACTTCAACTGAAACCGGTGCTATCCTCCTTTGGAAGTGGATCTCAGCCTTGA

**Table S1 | Candidate genes for the oxidation of sparteine to lupanine in NLL.** Organ-specific expression profile of the selected oxidases in a bitter NLL cultivar (Oskar) and a sweet cultivar (Tanjil).

| Gene ID from the NLL reference genome (NCBI) | Name abbreviation |  | Expression level in Tanjil (TPM) | | |  |  |  | Expression level in Oskar (TPM) | | | |  |  | Pearson's correlation coefficient (compared to LDC) |
| --- | --- | --- | --- | --- | --- | --- | --- | --- | --- | --- | --- | --- | --- | --- | --- |
|  |  | Root | Stem | Leaf | Flower | Seed | Root | Stem | Leaf | Pedicel | Flower | Mature  pericarp | Seed | Young pod |  |
| *LOC109327937* | *LDC* | 0.1 | 4.6 | 0.0 | 0.0 | 0.0 | 17.9 | 233.6 | 319.0 | 202.6 | 0.0 | 32.3 | 0.0 | 23.8 | 1 |
| *LOC109338642* | *CYP76E36* | 15.3 | 6.7 | 0.0 | 4.3 | 0.5 | 9.2 | 38.2 | 50.5 | 27.6 | 0.0 | 13.8 | 3.3 | 8.1 | 0.96 |
| *LOC109360201* | *CYP71D189* | 1.8 | 1.6 | 0.4 | 2.7 | 0.2 | 13.7 | 45.0 | 63.1 | 52.4 | 0.0 | 27.8 | 0.0 | 13.2 | 0.95 |
| *LOC109357725* | *CYP71A168* | 0.0 | 11.5 | 1.6 | 1.1 | 0.1 | 4.5 | 96.4 | 167.8 | 34.7 | 0.0 | 44.3 | 0.2 | 21.2 | 0.92 |
| *LOC109337773* | *SDR1* | 26.7 | 5.0 | 0.0 | 7.6 | 17.7 | 6.8 | 74.1 | 89.3 | 150.7 | 23.4 | 87.1 | 7.4 | 97.3 | 0.68 |

9

| **Table S2 \| List of DNA oligos used in this study.** | | | |
| --- | --- | --- | --- |
| **Name** | **Sequence** | **Target** | **Purpose** |
| mGFP5_pEAQ_FW | GGCTTAAUATGAGTAAAGGAGAAGAACTTTTC | *mGFP5* from the plasmid pCAMBIA1302 | USER cloning into pEAQ-USER |
| mGFP5_pEAQ_RV | GGTTTAAUTTATTTGTATAGTTCATCCATGCC | *mGFP5* from the plasmid pCAMBIA1302 | USER cloning into pEAQ-USER |
| CYP71D189_pEAQ_FW | GGCTTAAUATGGAGCTTCAAAACCCTTTC | *CYP71D189* from NLL *cultivar* Oskar | USER cloning into pEAQ-USER and genotyping of CYP71D189^KO^ NLL plants |
| CYP71D189_pEAQ_RV | GGTTTAAUTTAAGGCATACGAGTAACAATTGGA | *CYP71D189* from NLL *cultivar* Oskar | USER cloning into pEAQ-USER and genotyping of CYP71D189^KO^ NLL plants |
| SDR1_pEAQ_U_FW | GGCTTAAUATGGTAGAAACTACTTCCAACAACAG | *SDR1* from NLL *cultivar* Oskar | USER cloning into pEAQ-USER |
| SDR1_pEAQ_U_RV | GGTTTAAUTCATGGAGCAATATAGGAACCATCC | *SDR1* from NLL *cultivar* Oskar | USER cloning into pEAQ-USER |
| CYP76E36_pEAQ_FW | GGCTTAAUATGGATTATCTAACACTTTTTCTACTCA | *CYP76E36* from NLL *cultivar* Oskar | USER cloning into pEAQ-USER |
| CYP76E36_pEAQ_RV | GGTTTAAUTTATGCTTTGATAGGAATAACTAGGAGA | *CYP76E36* from NLL *cultivar* Oskar | USER cloning into pEAQ-USER |
| CYP71A168_pEAQ_FW | GGCTTAAUATGCCTCTTCTATCTATTCAAGAG | *CYP71A168* from NLL *cultivar* Oskar | USER cloning into pEAQ-USER |
| CYP71A168_pEAQ_RV | GGTTTAAUTCAAGGCTGAGATCCACTTC | *CYP71A168* from NLL *cultivar* Oskar | USER cloning into pEAQ-USER |
| CYP71D189_TaqMan_FW | ATATCAGCAATTGAGGAAGG | *CYP71D189* from NLL *cultivar* Oskar | Primer for TaqMan assay for genotyping the mutant NLL DNA library |
| CYP71D189_TaqMan_RV | TCAAGTTTAGCCTTTGTCTT | *CYP71D189* from NLL *cultivar* Oskar | Primer for TaqMan assay for genotyping the mutant NLL DNA library |
| CYP71D189WT_TaqMan_HEX | CAGGAGAACTATGGGTTAGT | *CYP71D189 ^WT^* allele from NLL *cultivar* Oskar | *CYP71D189 ^WT^* allele-specific probe for TaqMan assay containing a HEX fluorophore and a BHQ1 quencer |
| CYP71D189KO_TaqMan_FAM | CAGGAGAACTATAGGTTAGTGA | *CYP71D189 ^KO^* allele from NLL *cultivar* Oskar | *CYP71D189 ^KO^* allele-specific probe for TaqMan assay containing a FAM fluorophore and a BHQ1 quencer |
| pEAQ_Seq_FW | GCTTCTGTATATTCTGCCCAAATTCG | pEAQ-USER | Sanger sequencing of pEAQ-USER constructs and culture PCR |
| pEAQ_Seq_RV | CCGCTCACCAAACATAGAAATGC | pEAQ-USER | Sanger sequencing of pEAQ-USER constructs and culture PCR |

10

# References

Mancinotti, D., Czepiel, K., Taylor, J.L., Golshadi Galehshahi, H., Møller, L.A., Jensen, M.K., Motawia, M.S. *et al.* (2023) The causal mutation leading to sweetness in modern white lupin cultivars. *Sci. Adv.* 9, eadg8866.

11
